# Applications of near-infrared spectroscopy in neurocritical care

**DOI:** 10.1117/1.NPh.10.2.023522

**Published:** 2023-06-30

**Authors:** Rachel Thomas, Samuel S. Shin, Ramani Balu

**Affiliations:** a University of Pennsylvania, Department of Neurology, Philadelphia, Pennsylvania, United States; b Inova Fairfax Hospital, Medical Critical Care Service, Falls Church, Virginia, United States

**Keywords:** near-infrared spectroscopy, stroke, traumatic brain injury, cerebral autoregulation

## Abstract

**Significance:**

Acute brain injuries are commonly encountered in the intensive care unit. Alterations in cerebrovascular physiology triggered by the initial insult can lead to neurological worsening, further brain injury, and poor outcomes. Robust methods for assessing cerebrovascular physiology continuously at the bedside are limited.

**Aim:**

In this review, we aim to assess the potential of near-infrared spectroscopy (NIRS) as a bedside tool to monitor cerebrovascular physiology in critically ill patients with acute brain injury as well as those who are at high risk for developing brain injury.

**Approach:**

We first review basic principles of cerebral blood flow regulation and how these are altered after brain injury. We then discuss the potential role for NIRS in different acute brain injuries. We pay specific attention to the potential for NIRS to (1) identify new brain injuries and clinical worsening, (2) non-invasively measure intracranial pressure (ICP) and cerebral autoregulation, and (3) identify optimal blood pressure (BP) targets that may improve patient outcomes.

**Results:**

A growing body of work supports the use of NIRS in the care of brain injured patients. NIRS is routinely used during cardiac surgeries to identify acute neurologic events, and there is some evidence that treatment algorithms using cerebral oximetry may result in improved outcomes. In acute brain injury, NIRS can be used to measure autoregulation to identify an “optimum” BP where autoregulation status is best preserved. Finally, NIRS has been utilized to identify oximetry thresholds that correlate with poor outcome as well as identify new focal intracranial hemorrhages.

**Conclusions:**

NIRS is emerging as a tool that can non-invasively measure brain function in critically ill patients. Future work will be aimed at technical refinements to improve diagnostic accuracy, as well as larger scale clinical trials that can establish a definitive impact on patient outcomes.

## 1 Introduction

In the neurological intensive care unit (ICU), patients have pathophysiologic alterations in both systemic and intracranial physiology that require targeted interventions. Encased within the skull, diagnostic interrogations of brain physiology are more difficult compared to the rest of the body. The brain is particularly vulnerable to damage after ischemic and traumatic insults due to its high metabolic demands as well as the fact that it is enclosed within a rigid vault with little room to expand. Non-invasive, real-time monitors of brain function that can be deployed at the bedside are sorely needed. Near-infrared spectroscopy (NIRS) offers this possibility and has been studied in multiple forms of brain injury, including diffuse hypoxic-ischemic brain injury after cardiac arrest,^1^ subarachnoid hemorrhage (SAH),^2^^,^^3^ acute ischemic stroke,^4^^,^^5^ and traumatic brain injury (TBI).^6^^,^^7^ In this review, we will first discuss principles of cerebrovascular physiology and then discuss the features of and specific applications for NIRS in acute brain injuries.

## 2 Cerebral Blood Flow: Hemodynamic Autoregulation and Cerebrovascular Reactivity

The cerebrovascular network is structured to closely regulate cerebral blood flow (CBF) to ensure adequate perfusion. This is primarily controlled by vascular smooth muscle cells lining the pial and intraparenchymal arteries, which alter the vessel diameter and thus cerebrovascular resistance (CVR). Multiple mechanisms exist to modulate resistance and therefore CBF, including hemodynamic (myogenic) autoregulation and cerebrovascular carbon dioxide (${\mathrm{CO}}_{2}$) reactivity. When these mechanisms are intact, the brain is adequately perfused and metabolic homeostasis is achieved. However, when neurovascular injury is sustained, these systems fail and subsequent vascular and metabolic derangements ensue. These derangements can lead to mismatches between cerebral metabolic demand and energy supply, ultimately causing further secondary brain injury. A major need in the ICU, therefore, is the ability to detect these vascular changes, and thereby make meaningful interventions to preserve and optimize cerebral hemodynamics.

### 2.1 Hemodynamic Autoregulation: Blood Pressure and Cerebral Perfusion

**Fig. 1:**
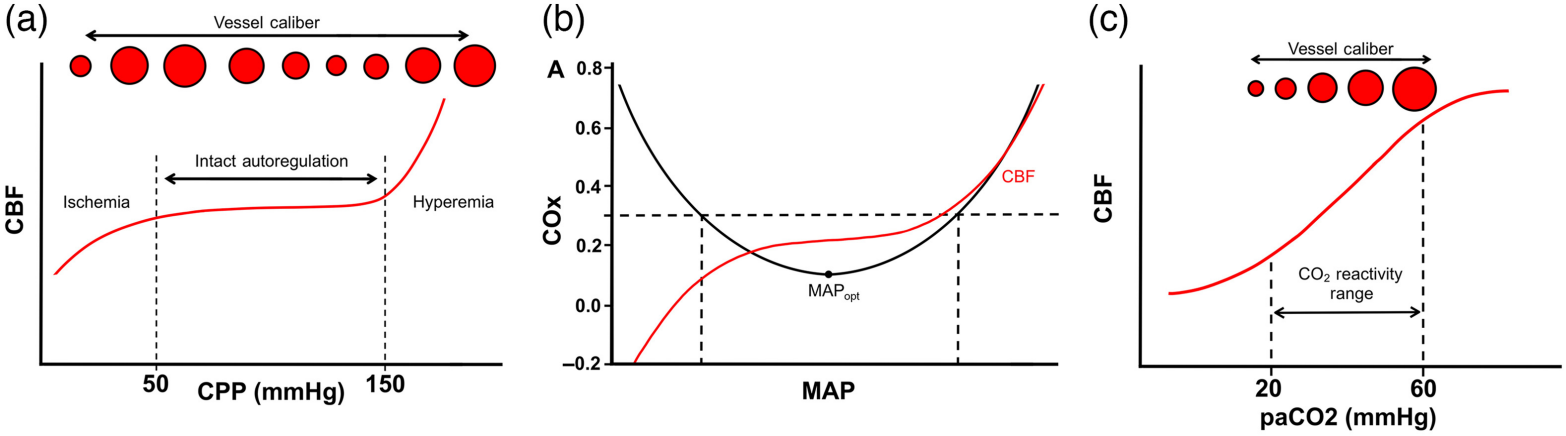
Major concepts in cerebral autoregulation and vascular reactivity. (a) Graph depicting relationship of CPP to CBF with intact cerebral autoregulation. Within the zone of intact autoregulation (dashed lines), changes in vessel diameter maintain consistent CBF. When CPP drops below the zone of autoregulation, ischemia ensues. Conversely, elevated pressures above the autoregulation zone lead to hyperemia and edema. (b) Optimal MAP (${\mathrm{MAP}}_{\mathrm{opt}}$) is calculated by comparing the cerebral TOI as measured by NIRS across the patient’s range of BP. The autoregulatory curve is superimposed in red, and the gray box denotes the patient’s MAP range that corresponds to those that fall within preserved autoregulation status (COx≤0.3). ${\mathrm{MAP}}_{\mathrm{opt}}$ corresponds to the nadir of the COx curve. (c) Cerebrovascular reactivity to ${\mathrm{CO}}_{2}$ is depicted with lower ${\mathrm{paCO}}_{2}$ causing vasoconstriction.

The cerebral vasculature reacts to changes in mean arterial pressure (MAP)—which ultimately affects vessel transmural pressure—by altering vessel diameter to regulate CBF.^8^^,^^9^ Changes in vessel diameter can alter intracranial pressure (ICP) through changes in cerebral blood volume, which ultimately can impact cerebral perfusion pressure (CPP). CPP is the major pressure gradient driving CBF (and therefore oxygen delivery) to cerebral tissue and can be crudely estimated by the equation CPP = MAP – ICP.^10^ [Fig. 1(a)]. It should be noted, however, that a more complete model requires consideration of the effects of vessel wall tension/tone^11^^,^^12^ in addition to ICP, on CPP. A more accurate equation for actual CPP (aCPP) is aCPP = MAP − CrCP, where CrCP is the “critical closing pressure.” CrCP is the arterial blood pressure at which CBF ceases due to its inability to overcome ICP and active wall tension in the small arterioles.^13^ CrCP can calculated by the linear slope of plotting MAP and CBF velocity and extrapolating the pressure at which velocity = 0.^14^

When autoregulatory mechanisms are intact, increases in CPP (caused either by increases in mean arterial blood pressure or decreases in ICP) are counteracted by vasoconstriction through the myogenic reflex, increasing CVR to maintain consistent CBF^15^ (Fig. 1). However, this commonly accepted heuristic may be an oversimplified view, given the wide individual differences in autoregulatory ranges as well as significant effects of sedative, anesthetic, and cardiovascular medications on autoregulation.^16^

In theory, autoregulation status can be measured by calculating the correlation between pressure (either CPP or MAP) and CBF over a range of spontaneously fluctuating pressures (positive correlation implies disrupted autoregulation, whereas absent correlation implies intact autoregulation). The correlation between MAP and ICP, a surrogate estimate of cerebral autoregulation, termed the cerebrovascular pressure reactivity index (PRx) has been validated as a marker of outcome in patients with multiple forms of brain injury.^17^ As a correlation coefficient, PRx values lie between −1 and +1. Values closer to +1 suggest worsening autoregulation status. PRx can be calculated over serial, overlapping time windows to provide a near real-time estimate of autoregulation status that is updated continuously. PRx values >0.3 are generally interpreted to signify impaired autoregulation.^18^ Although PRx can provide valuable, clinically actionable data on autoregulation status, it is limited to patients receiving invasive ICP neuromonitoring. Transcranial Doppler (TCD) measures of CBF velocity can be recorded non-invasively; however, the use of TCD for continuous autoregulation measurement is practically hindered by the difficulty in performing continuous TCD recordings. Because of these issues, NIRS has emerged as an important tool for non-invasive bedside autoregulation assessment in the ICU. There are several NIRS-derived values that have been utilized to assess cerebral autoregulation. Early studies investigated the correlation between fluctuations in MAP and the difference between oxygenated and deoxygenated hemoglobin ([HbD] = [HbO] − [HbR]).^19^ This relative value is highly susceptible to artifacts.^20^ Alternative measures include (1) tissue oxygenation index (TOI), or the correlation between MAP and NIRS-derived measures of oxygenation calculated by the ratio of HbO/(HbO + HbR) multiplied by a scaling factor and expressed as a percentage and (2) cerebral oximetry index (COx), which is the correlation between MAP and NIRS-derived regional cerebral oxygen saturation (${\mathrm{rSO}}_{2}$).^20^ These measures are less susceptible to movement artifacts. All these parameters function similarly to PRx in that the more negative the relationship between changes in MAP and measures of oxygenation are, the more intact the autoregulation is assumed to be.^21^ Alternatively, NIRS studies have also leveraged the presence of baseline low frequency oscillations in arterial blood pressure (ABP) that demonstrate NIRS correlates in the forms of oscillating changes to [HbO] and [HbR]; acute brain injuries (ABIs) have been shown to alter the oscillatory pattern and are thought to represent altered autoregulation.^22^ These approaches have been used to measure autoregulation status in patients with TBI,^21^ ischemic stroke,^22^^–^^24^ SAH,^25^ and diffuse hypoxic ischemic brain injury after cardiac arrest.^26^^–^^28^

Diffuse correlation spectroscopy (DCS) is a non-invasive diffuse optical technique that can provide a measure of relative CBF (rCBF) changes through detecting fluctuations in light intensity caused by red blood cells in the microvasculature (see Sec. 3.3), which allows for a direct comparison of ICP/CPP with CBF.^29^

**Fig. 2:**
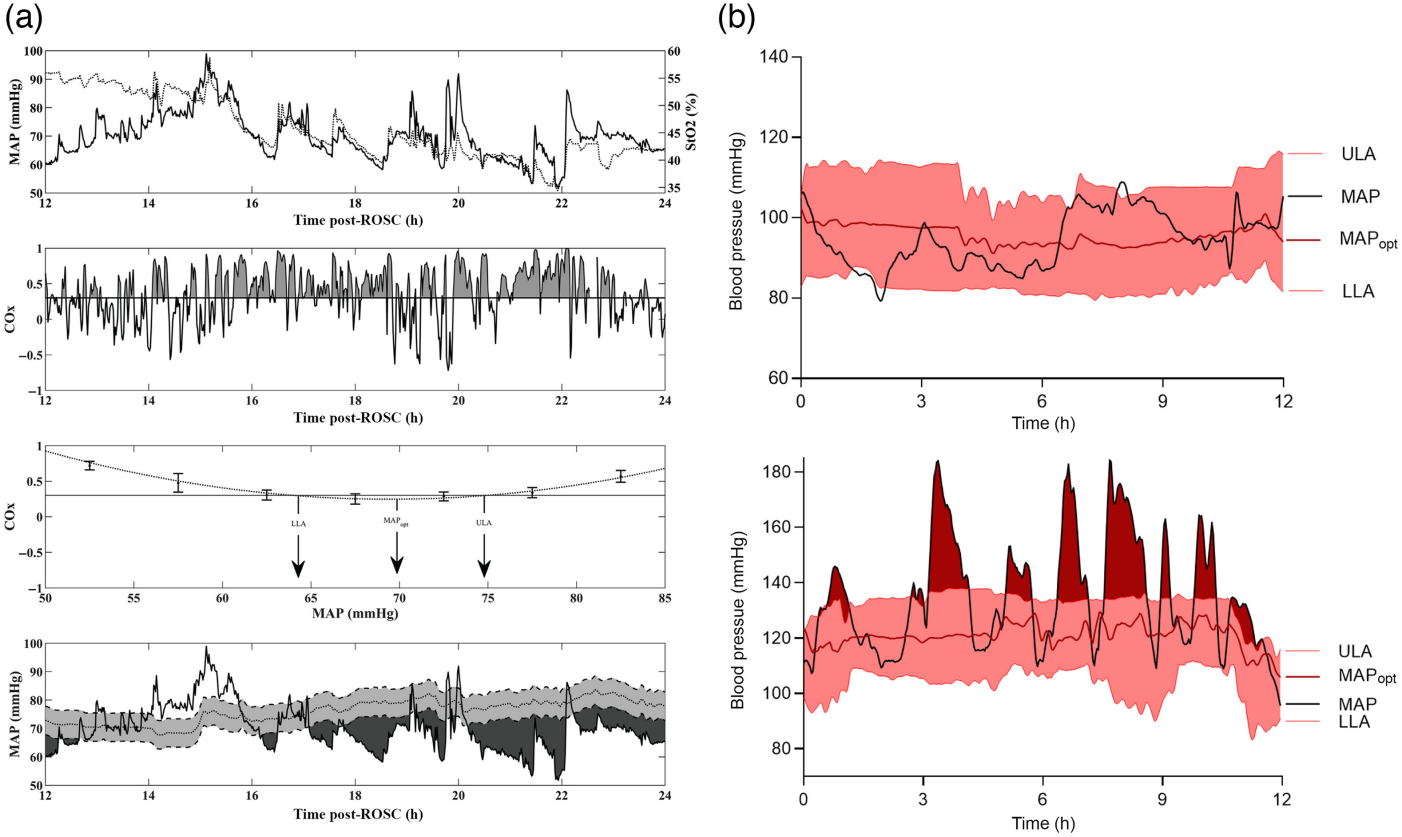
Examples of using NIRS derived autoregulation indices to identify ${\mathrm{MAP}}_{\mathrm{opt}}$. (a) Data from a pediatric cardiac arrest patient. Top graph shows the time course of fluctuations in MAP and $S_{t}O_{2}$ over a 24-h period. Second from top, temporal fluctuations in COx are shown. Periods of time with poor autoregulation (i.e., COx>0.3) are shaded gray. Second from bottom, method for deriving ${\mathrm{MAP}}_{\mathrm{opt}}$ (MAP where COx is minimized) and both ULA and LLA (BP where COx > 0.3) is shown. Bottom graph shows temporal trends in ${\mathrm{MAP}}_{\mathrm{opt}}$ superimposed on top of the patient’s actual BP. Areas shaded dark gray represent periods of time when actual MAP was significantly below ${\mathrm{MAP}}_{\mathrm{opt}}$ (defined as periods where $\mathrm{MAP}<{\mathrm{MAP}}_{\mathrm{opt}}-5\;\mathrm{mmHg}$). (b) Similar data from adult patients with non-traumatic subarachnoid hemorrhage. Black trace represents patient’s actual MAP, red trace represents trends in ${\mathrm{MAP}}_{\mathrm{opt}}$ calculated from COx. Shaded red areas represent the range of intact autoregulation. The top curve shows a patient with BP trends where MAP largely stayed within ULA and LLA, whereas bottom curve shows a patient where BP often exceeded the ULA. [(a)- taken from Ref. 30 and (b)- taken from Ref. 31].

In addition to measuring autoregulation status, NIRS can be utilized to identify an optimal MAP (${\mathrm{MAP}}_{\text{opt}}$) or optimal CPP (${\mathrm{CPP}}_{\mathrm{opt}}$) where autoregulation status is ideal [Fig. 1(b)].^18^ Maintaining ${\mathrm{MAP}}_{\mathrm{opt}}$ or ${\mathrm{CPP}}_{\mathrm{opt}}$ could potentially limit secondary injury that occurs due to deviations in blood pressure (BP) that occur outside the range of optimal autoregulation. The general strategy is to serially measure an NIRS-based oxygenation index, such as COx or TOI, as well as a measure of perfusion, MAP or CPP. The blood/perfusion pressure where COx is at a minimum is taken to be ${\mathrm{MAP}}_{\mathrm{opt}}$ (Fig. 2) or ${\mathrm{CPP}}_{\mathrm{opt}}$. A recent study investigated this strategy in patients with a variety of ABIs and found that greater absolute difference between clinically observed and optimal MAP was associated with increased risk of death.^32^

### 2.2 Cerebrovascular Reactivity: ${\mathrm{CO}}_{2}$-Regulated Vascular Changes

Chemoreceptors in the cerebral vasculature induce vasodilation in response to increases in the partial pressure of ${\mathrm{CO}}_{2}$ (${\mathrm{pCO}}_{2}$).^33^ This is thought to be related to ${\mathrm{pCO}}_{2}$-triggered release of vasoactive factors, such as nitric oxide, endothelin-1, and endothelial-derived hyperpolarizing factor from the vascular endothelium itself, and can be independent of changes in pH.^34^ Hyperventilation (which decreases ${\mathrm{pCO}}_{2}$) constricts cerebral blood vessels and concomitantly can decrease ICP due to reductions in cerebral blood volume [Fig. 1(c)]. Cerebrovascular reactivity is compromised after brain injury, leading to patchy, asymmetric patterns of dilation in response to hypercapnic stimuli in which the healthy vessels dilate more readily, leading to preferentially greater regional CBF.^35^

Alterations in cerebrovascular reactivity are common in acute brain injury including TBI,^35^^,^^36^ SAH,^37^ and stroke,^38^^,^^39^ and have been shown to be poor prognostic indicators.^37^^,^^40^ Although blood oxygen level dependent functional magnetic resonance imaging (BOLD-fMRI) has been used previously to study cerebrovascular reactivity, magnetic resonance imaging (MRI) is a resource-intensive tool requiring significant time, support staff availability, and maintenance costs. Non-invasive assessment of cerebral autoregulation has been performed using TCDs in both healthy and critically ill patients by studying its relationship to systemic and respiratory CO2 levels.^41^^,^^42^ Specifically, different responses between end-tidal ${\mathrm{CO}}_{2}$ (${\mathrm{ETCO}}_{2}$) levels to posterior cerebral artery and vertebral artery blood flow (BF) during exercise have been demonstrated,^41^ sugggesting that ${\mathrm{CO}}_{2}$ may have unique effects for different vessels throughout the brain. However, TCDs are limited by dependence on the technologist and difficulty with maintaining prolonged, continuous measurements. NIRS, in addition to its relatively low cost and portability, also demonstrates higher temporal resolution compared to functional MRI (fMRI) (albeit at the cost of spatial resolution) and can provide continuous assessments. Experimental animal studies using NIRS can directly show a significant correlation between the level of pCO2 changes and CBF.^43^ In addition, using NIRS in humans, a cerebrovascular reactivity index can be calculated by calculating the absolute change in HbO, HbR, and total hemoglobin (Hb)^36^^,^^44^^,^^45^ or alternatively ${\mathrm{rSO}}_{2}$^45^^,^^46^ per 1 kPa increase in ${\mathrm{ETCO}}_{2}$. ${\mathrm{CO}}_{2}$ reactivity measurements via fNIRS have been validated against both TCD^44^ and BOLD-fMRI,^36^^,^^45^ and demonstrate a significantly high correlation. Prior studies have utilized NIRS following various forms of both acute and chronic brain injury,^47^^,^^48^ as discussed further in each disease-specific application subsection below. ${\mathrm{CO}}_{2}$ levels can also influence autoregulation,^49^ and this interplay may be dysregulated in pathological states, such as sepsis.^42^

### 2.3 Neurovascular Coupling: Neural Activity-Induced Vascular Control

With intact neurovascular coupling, cerebral blood vessels dilate in response to increased local energy or metabolic demands.^50^^,^^51^ Neurovascular coupling relies on neuronal activity-dependent release of chemical signals, such as prostaglandins, nitric oxide, and adenosine,^51^ to regulate and distribute CBF to the regions of highest neural activity. Because local BF responses during neurovascular coupling exceed metabolic demand, functional NIRS (fNIRS) can detect neurovascular coupling as a transient increase in both oxy- and total hemoglobin.^52^^,^^53^ ABIs can trigger inverse neurovascular coupling, where higher metabolic demand results in paradoxical vasoconstriction, depriving metabolically active brain tissue of oxygen and glucose delivery and potentiating neurotoxicity.^54^^,^^55^ This is most notably seen in association with cortical spreading depolarizations (CSDs), which are pathologic waves of extreme depolarization that emanate from areas of acute brain injury (see Sec. 5.3).^55^

## 3 Advantages and Disadvantages of NIRS and Related Technologies in the ICU

The limited absorption of near-infrared (NIR) range (650 to 950 nm) light through the skull provides the ability to non-invasively detect changes in hemodynamics and oxygenation in the brain tissue lying underneath. In addition, given its potential portability and high temporal resolution, diffuse optical imaging is potentially ideal for providing real-time measurements of cerebral physiology at the bedside. Multiple subcategories of non-invasive optical imaging provide distinct advantages and disadvantages, as discussed below (Table 1).

**Table 1:** Comparison of different NIRS methods and related technologies.

NIRS modality	Principles used	Information provided	Applications/advantages	Disadvantages
CW-NIRS	Constant illumination	Relative changes in [HbO] and [HbR]	Continuous monitoring for relative changes in CBF in response to discrete clinical events/interventions	Only relative changes
	Measures changes in NIR absorption of HbO and HbR using MBLL			Susceptible to background light contamination
			Commercial devices available for clinical use	
			Easily portable	
FD-NIRS	Intensity-modulated illumination	Absolute [HbO], [HbR], and ${\mathrm{rSO}}_{2}$	Compare absolute hemodynamic and oxygenation changes,	Bulky size/expensive
				Lack of commercial devices
	Phase-resolved detection		Make comparisons within patients over time and between patients	
	Measures the average intensity of the detected light as well as fluctuating intensity and phase shift		Less susceptible to background light artifact	
TD-NIRS	Extremely short, pulsed illumination	Absolute [HbO], [HbR], and ${\mathrm{rSO}}_{2}$	Compare absolute hemodynamic and oxygenation changes	Bulky/expensive
				Lack of commercial devices
	Time-resolved detection of emerging photons (time of flight [TOF])	Detailed assessment of the tissue’s optical properties	Make comparisons within and between-patients	
DCS	Coherent NIR-range light	Relative changes in CBF	Detecting changes in CBF and cerebral ischemia	Can only measure relative changes
	Detects fluctuations in light intensity over time produced by scattering particles			
				Susceptible to motion and background light artifacts
				Paucity of commercially available devices
DCS/NIRS	Hybrid of DCS and NIRS	Concurrent CBF, [HbO], [HbR], and ${\mathrm{rSO}}_{2}$	Detection of cerebral ischemia	Repeated calibration with tracer injection if using CW-NIRS
			Can detect absolute changes in CBF if using contrast enhanced NIRS	
			Can monitor cerebral oxygen metabolism	Bulky/expensive
				Lack of commercially available devices
DCT	Analyzes CBF data from DCS using many source-detector pairs and a wide FOV	Dynamic, 3D imaging of CBF	Obtaining spatial information in focal injuries	Susceptible to noise, artifacts
				Decreased penetration to deeper tissues

### 3.1 Continuous Wave-NIRS

Continuous wave-NIRS (CW-NIRS) is the most used technology in commercial NIRS devices and in the ICU. It utilizes constant illumination of infrared light and measures changes in the absorption of the light that passes through the tissue to derive changes in HbO and HbR concentrations using the Modified Beer-Lambert Law (MBLL). CW-NIRS provides a measure of relative changes in hemodynamics and oxygenation and cannot quantify absolute HbO, HbR, and HbT concentrations.^56^^,^^57^ However, the portable nature and commercial availability of CW-NIRS systems makes them very attractive candidate devices for use at the bedside in the neonatal ICU (NICU). Commercial CW-NIRS devices generally utilize a multi-distance approach in which light is detected at ≥2 different distances from the source to allow separation of superficial (e.g., scalp) signals from deeper signals in the brain.^56^

### 3.2 Frequency Domain-NIRS and Time Domain-NIRS

Frequency domain (FD-)NIRS employs intensity-modulated illumination (∼100  mHz frequency) and phase-resolved detection, which measures not only the average intensity of the detected light but also its fluctuating amplitude and phase shift (φ).^56^^,^^58^ One important adantage of FD-NIRS is that it can provide quantitative measures of absolute HbO, HbR, and HbT as well as tissue oxygen status (${\mathrm{rSO}}_{2}$, see Sec. 5.2 for clinical importance). Absolute measures allow better ability to compare data longitudinally within-subjects as well as between-subjects. In addition, at the modulation frequencies typically used in FD-NIRS (<150  MHz), recorded signals are less susceptible to ambient light, minimizing background artifacts.^57^^,^^59^^,^^60^ This may be particularly advantageous in the ICU where there are numerous potential artifacts from room lighting and stray light from other devices. In addition, the phase shift data derived from FD-NIRS also help to improve the signal-to-noise ratio as well as to improve depth sensitivity in cerebral tissue.^61^ The disadvantage to this technology is its large size, high cost, and paucity of commercially-available devices.

Time domain (TD-)NIRS in contrast uses pulsed illumination and time-resolved detection. TD-NIRS devices emit an extremely short (≤100  ps), concentrated pulse of light into the tissue and measures the arrival times and paths of the photons that emerge from the tissue. The temporal distribution of the detected photons allows an assessment of the tissue’s optical properties (scattering and absorption); therefore, this technique also provides absolute hemodynamic/oxygenation data.^56^ This technology yields the highest amount of information, but it is also the most complex technology both for operation and analysis. As with FD-NIRS, there is a paucity of commercially available TD-NIRS devices approved for use in clinical settings, though at least one company has developed a portable bedside device that is similar in size to commercially available CW-NIRS.^62^

### 3.3 Diffuse Correlation Spectroscopy

DCS is a related technology that uses coherent NIR-range light to measure hemodynamic changes in deep tissue microvasculature. It detects fluctuations in light intensity over time produced by mobile sources of scatter (primarily RBCs in the microvasculature) to produce a measure of rCBF known as a BF index (BFI),^29^ which is proportional to absolute CBF.^63^^,^^64^ It has excellent temporal resolution and penetrates through superficial tissue layers.^65^ DCS has been combined with NIRS in hybrid instruments to allow for absolute CBF measures, with NIRS used to calculate the DCS calibration coefficient γ, a constant that is dependent on tissue factors including the geometry and composition of regional microvasculature (e.g., arterial versus capillary versus venule) as well as tissue heterogeneity and optical properties, with tissue BF=γ BFI.^63^^,^^64^ Improvement in determination of γ can be achieved by contrast-enhanced NIRS using injections of indocyanine green (ICG), a light-absorbing optical dye, and measuring its time-dependent absorption signal through tissue to provide absolute CBF at any given time point. The requirement of repeated ICG injections limits its use for continuous monitoring, and therefore other studies have created individualized patient calibration protocols to minimize this need.^65^ In addition, hybrid DCS/NIRS devices can simultaneously measure CBF and oxy-deoxy-hemoglobin concentrations, respectively,^66^^–^^68^ which allows for the derivation of metabolic rate of tissue oxygen consumption and evaluation of possible tissue ischemia (see Sec. 5.2). DCS has been validated against numerous gold-standard CBF techniques, including arterial spin labeling fMRI,^69^ TCD,^70^ and xenon-enhanced computed tomography.^67^ An advantage over these technologies in that DCS provides a continuous bedside assessment of absolute CBF. A disadvantage of DCS is the limited number of commercially available devices with no device currently cleared for clinical use. However, at least two bedside hybrid systems, one with DCS coupled with FD-NIRS^71^ and another with TD-NIRS,^68^ have become commercially available for research over the past few years.

#### 3.3.1 Diffuse correlation tomography

While NIRS offers superior temporal resolution, a major limitation of NIRS is its poor spatial resolution. This presents a challenge for ABIs that classically involve more focal areas of injury. One advancement in DCS technology is diffuse correlation tomography (DCT), which can generate three-dimensional (3D) images of BF by analyzing CBF data from DCS using many source-detector pairs and a wide field of view (FOV) for image reconstruction. There are multiple approaches to DCT analysis (traditional versus speckle contrast), which are beyond the scope of this paper.^72^ Numerous animal studies have focused on the application of DCT to cerebral physiology, including in pharmacologically-induced CSDs,^73^ which are pathologic sequelae of many type of ABIs (see Sec. 5.3) and ischemia/stroke.^74^^,^^75^ This technology is limited by its susceptibility to noise and difficulty penetrating deeper tissues as would be required for CBF monitoring in humans.^72^ Moreover, speckle contrast DCT in particular requires a window (e.g., burr hole or craniotomy) for intracerebral monitoring.^73^

## 4 Applications of NIRS in Specific Brain Injuries

Given the often profound impact of ABIs upon cerebral hemodynamics, NIRS and related technologies are powerful tools for assessment of patients’ cerebral autoregulation, vascular reactivity, and tissue oxygenation. To this end, many studies have utilized optical imaging to both diagnose focal/global injuries as well as to provide real-time data for optimizing clinical management (e.g., optimizing CPP). Below, we will discuss applications based upon particular forms of acute brain injury.

### 4.1 Sudden Cardiac Arrest

Outcomes in survivors of sudden cardiac arrest are largely driven by the burden of hypoxic ischemic brain injury.^76^ Early studies using NIRS showed that a cerebral ${\mathrm{rSO}}_{2}$ reduction of 20% or a level below 50% to 60% during induced ventricular fibrillation in humans is associated with ischemic brain injury.^30^ Cerebral oximetry has since been studied during both the initial resuscitation period as well as during subsequent post-arrest ICU care. In both adults and children, higher ${\mathrm{rSO}}_{2}$ values measured during resuscitation are associated with higher rates of return of spontaneous circulation.^77^^–^^79^ One study in adults suggested that higher intra-arrest ${\mathrm{rSO}}_{2}$ may also be associated with improved neurologic outcome.^79^

In the post-arrest period, NIRS has been utilized to measure the association between cerebral autoregulation state and neurologic outcome as well as to identify the ${\mathrm{MAP}}_{\mathrm{opt}}$. Studies in both adults and children have established the feasibility of using NIRS to identify individualized ${\mathrm{MAP}}_{\mathrm{opt}}$, which is generally higher than targets recommended in post-arrest care guidelines [Fig. 2(a)].^26^^–^^28^^,^^80^^,^^81^ In children, the overall burden of deviations in BP below ${\mathrm{MAP}}_{\mathrm{opt}}$ is associated with increased risk of mortality.^80^

### 4.2 Extracorporeal Life Support

Extracorporeal life support is an important therapy used for high-risk cardiac surgeries [cardiopulmonary bypass (CPB)] as well as longer term support of patients with severe cardio-respiratory failure in the ICU [extracorporeal membrane oxygenation (ECMO)]. ABIs are common during CPB and ECMO due to both the high risk of thrombus formation within the extracorporeal circuit (which can lead to ischemic stroke) as well as the need for systemic anticoagulation (which can result in intracranial hemorrhage). NIRS-based cerebral oximetry has become a standard practice for monitoring brain function during open cardiac surgeries where CPB is used. Algorithms to optimize ${\mathrm{rSO}}_{2}$ during surgery have been developed^82^ and shown to be feasible in reversing episodes of cerebral desaturation.^1^^,^^83^ In adults, a recent trial showed that using a strategy to optimize ${\mathrm{rSO}}_{2}$ was associated with improved post-operative memory function; however, there was no difference in the burden of cerebral hypoxia between control and intervention groups.^84^ It remains unclear what aspects of the cerebral optimization strategy were responsible for the difference. In patients receiving ECMO, decreases in ${\mathrm{rSO}}_{2}$ are associated with the development of ABIs (both acute ischemic strokes and intracranial hemorrhage)^85^^,^^86^ and worse outcomes.^87^ Further work is needed, however, to determine if cerebral oximetry monitoring is associated with improved outcomes.

### 4.3 Non-Traumatic Subarachnoid Hemorrhage

Non-traumatic SAH most often occurs as the result of the spontaneous rupture of a cerebral aneurysm.^88^ In addition to the primary brain injury caused by the initial bleeding event, SAH can trigger delayed cerebral ischemia (DCI) in the days that follow DCI. Multiple mechanisms—including cerebral vasospasm, microthrombosis, and CSD—mediate DCI, resulting in new ischemic infarcts that can occur anywhere between 3 and 4 days to more than 2 weeks after aneurysm rupture.^89^^,^^90^ TCD is routinely used clinically to screen for DCI but has only modest sensitivity and specificity.^31^^,^^91^

Studies have evaluated the ability of NIRS to identify both DCI (defined clinically and/or radiographically) as well as cerebral vasospasm (defined angiographically) after subarachnoid hemorrhage. Using TD-NIRS, one study showed acute drops in both ${\mathrm{rSO}}_{2}$ and oxy-hemoglobin in eight patients, six of which had angiographically confirmed vasospasm.^2^ Another more recent study used CW-NIRS as a marker of DCI (defined clinically as a sustained decrease in Glasgow Coma Scale score of >2 points or new focal neurologic deficit without other explainable cause) and showed that a reduction in ${\mathrm{rSO}}_{2}$ of >14.7% from baseline had 85.7% sensitivity and specificity for detecting DCI.^92^ In both studies, the accuracy of NIRS was superior to TCD.

DCI is often preceded by impaired cerebral autoregulation that can be identified using NIRS or TCD days before onset.^3^^,^^93^ A recent study used both NIRS (COx) and ICP (PRx) based continuous autoregulation measurements to calculate optimal MAP and found that the burden of deviations away from individualized ${\mathrm{MAP}}_{\mathrm{opt}}$ targets was associated with worse outcome [Fig. 2(b)].^94^ There was strong agreement between both COx and PRx based ${\mathrm{MAP}}_{\mathrm{opt}}$ values.

### 4.4 Acute Ischemic Stroke

Ischemic stroke occurs after occlusion of a cerebral artery with resultant cessation of blood flow. Treatment revolves around re-opening the occlusion (either with pharmacologic thrombolysis, catheter directed endovascular thrombectomy, or a combination) in suitable candidates and optimizing cerebral perfusion to prevent infarct growth while limiting hemorrhagic complications in all patients. The traditional configuration of NIRS using forehead sensors monitors oxygenation in brain tissue supplied by the middle and anterior cerebral arteries. For this reason, NIRS has been studied during early revascularization therapies as a tool to monitor therapeutic success as well as during the subsequent stages of care.

An observational study in 43 patients who received endovascular thrombectomy identified multiple types of desaturation events of potential clinical importance.^4^ Eleven patients showed distinct bilateral ${\mathrm{rSO}}_{2}$ drops during endotracheal intubation. During the intervention, small peaks (more common) as well as sustained rises (less common) in ${\mathrm{rSO}}_{2}$ were observed that correlated with recanalization. A greater interhemispheric ${\mathrm{rSO}}_{2}$ difference at the end of the case was associated with mortality. A more recent study showed that successful recanalization was associated with a significant reduction in interhemispheric ${\mathrm{rSO}}_{2}$ difference.^5^

NIRS has also been utilized to identify optimal MAP in the post-revascularization period using cerebral autoregulation measurements. During this time period, cerebral autoregulation is impaired, and vulnerable brain tissue surrounding the core infarct (termed the ischemic penumbra) is at risk for further ischemic damage (leading to expansion of total infarct volume) from hypoperfusion as well as hemorrhage from hyper/reperfusion injury. Petersen et al.^95^^,^^96^ calculated ${\mathrm{MAP}}_{\mathrm{opt}}$ as well as upper and lower limits of autoregulation (ULA and LLA, respectively) and showed that that the burden of time spent above ULA had worse outcomes and higher risk of hemorrhagic transformation. Future trials are required to evaluate whether targeting ${\mathrm{MAP}}_{\mathrm{opt}}$ and minimizing time spent above ULA lead to improved outcomes.

### 4.5 Traumatic Brain Injury

TBI is a leading cause of death and disability worldwide.^97^ Computed tomography (CT) imaging is the most frequently used diagnostic tool. Traumatic cerebrovascular injury, including SAH, subdural hematoma, epidural hematoma, and contusion/intraparenchymal hemorrhage, is often seen on CT, and the presence of different focal injuries can markedly affect management decisions. However, in low-resource, pre-hospital, and military/wartime settings, CT imaging is not readily available.

Due to the resulting increased absorption of NIR light by dense areas of extravascular blood, NIRS can be potentially used for focal vascular injury detection. CW-NIRS allows relative Hb concentrations to be determined, with a comparison to the contralateral hemisphere or nearby “healthy” tissue.^98^ Lower scattering/high absorbance occurs in areas of hematoma. The commercially available Infrascanner™ is a portable, handheld device that uses this principle to detect hematomas >3.5 cc in volume and <2.5  cm from the pial surface. Studies demonstrate a sensitivity of hematoma detection between 78% and 93% and specificity 82.9% and 90% with positive predictive value (PPV) of 77% and negative predictive value (NPV) of 90%.^6^^,^^7^ An alternative device (CrainScan, BYTEC, Germany) that also uses CW-NIRS found similar efficacy in hematoma identification in a cohort of 148 patients. Out of the 54 CT-confirmed hematoma cases, NIRS detected 48 with a sensitivity of 88.9%, specificity 77.7%, PPV 69.6%, and NPV 62.8%.^99^ A theme throughout these studies is that due to the limitations of NIRS in penetrating far into the brain, more deeply seated lesions, such as those in the posterior fossa, remain occult. In addition, bilateral and small lesions also pose a challenge. FD-NIRS can be coupled with multiple optodes to create a 3D reconstruction of the target area, allowing localization of the lesion within a centimeter. This FD-diffuse optical tomography offers the potential for effective pre-hospitalization diagnosis and triage based on non-invasive imaging.^100^ However, they are larger in size, limiting their portability.^98^

In addition to focal lesions in TBI, more widespread microvascular injury is quite common and contributes to secondary injury. CPP optimization may limit the secondary damage that occurs from diffuse microvascular injury. Zwiefel and colleagues compared autoregulation indices measured by invasive ICP monitoring (PRx) and NIRS [total hemoglobin index (THx)] in 40 hospitalized subjects with TBI and found moderate overall correlation between the two indices (r=0.56, p=0.0002) that improved (r=0.65) after removing patients with frontal hematomas. Across all patients, ${\mathrm{CPP}}_{\mathrm{opt}}$ and ${\mathrm{MAP}}_{\mathrm{opt}}$ values were similar when calculated using PRx and THx.^21^ This paves the way for eventual non-invasive individualized optimization of cerebral hemodynamics, which may improve long-term patient outcomes.

Finally, traumatic microvascular injury in TBI can lead to long term alterations in cerebrovascular reactivity to carbon dioxide. Amayot et al. explored the long-term effects of TBI on ${\mathrm{CO}}_{2}$ reactivity using a hypercapnia challenge using both BOLD-fMRI and NIRS. They found that in chronic TBI subjects, cerebrovascular reactivity was reduced both globally and to a greater extent focally (frontal regions) using both methods when compared to healthy controls.^36^

## 5 Challenges and Future Applications

### 5.1 Non-Invasive Measurement of ICP

Given that the skull is a rigid container, the pressure within (ICP) is equal to the sum of the pressures exerted by its contents, which include the cerebral vasculature, brain parenchyma, and the cerebrospinal fluid (CSF).^101^ An increase in volume to any of these components will raise ICP, and if ICP surpasses critical values the risk of cerebral herniation markedly increases.^101^ In addition, since ICP is a key determinant of CPP, its continuous measurement is clinically valuable.^15^ ICP monitoring in general requires the placement of invasive probes directly into brain tissue, which requires neurosurgical expertise and carries the risk of associated infection and hemorrhage.^102^ Many patients cannot have invasive monitors placed due to higher risks of bleeding. Therfore, a non-invasive means of determining a patient’s ICP would have substantial clinical benefit. TCD^103^ and optic nerve sheath ultrasound^104^ have been explored as non-invasive methods to estimate ICP, but in both cases results can be highly variable and are examiner dependent.^105^ Likewise, imaging approaches with CT and MRI have not provided reliable estimates of ICP and are limited in feasbility due to radiation exposure and cost/time, respectively.^105^

Diffuse optics methods (including NIRS and DCS) may provide a viable non-invasive method for ICP measurement. Early studies have examined their use in non-human primate (NHP) models^106^^,^^107^ as well as in infants,^108^ by estimating ICP from the derived cardiac waveform^106^ and comparing this to the gold-standard invasive monitors. Importantly, alterations to relative [HbO] alone (which can be obtained with basic NIRS devices) appear to change with ICP and when used in combination with machine learning, can be used to derive ICP from waveform features in NHP with validation against invasive neuromonitors and CBF data from DCS.^109^

A different approach is to utilize DCS to measure CrCP, which is proportional to ICP but also factors in vasomotor tone. Although TCD has been shown to be capable of measuring CrCP non-invasively,^110^ it is limited by the ability to provide continuous measurements, technical anatomic challenges, and the risk of confounding hemodnamic facors, such as turbulent flow in large insonnated arteries.^111^ Baker et al.^13^ have demonstrated the ability to optically measure CrCP using DCS, thus bypassing these limitations. By assessing pulsatile CBF waveforms through the microvasculature, and comparing this to pulsatile peripheral arterial BP waveforms, DCS provided a highly reproducible, accurate method for measuring CrCP and thereby aCPP in healthy adults, which was validated against TCD.^13^ These initial results are promising and future studies in brain injured patient populations have the potential to lead to the development of powerful ICU tools.

### 5.2 Detection of Cerebral Ischemia

Timely bedside identification of cerebral ischemia is a key principle in neurocritical care.^112^ This often requires placement of invasive, intraparenchymal monitors for measuring ICP, brain tissue oxygen tension (${\mathrm{PbtO}}_{2}$), CBF, and cerebral biochemistry. In general, invasive monitoring measures only surrogates for true ischemic injury and only samples small areas of injured tissue. In addition, using guideline endorsed physiological targets (e.g., ${\mathrm{PbtO}}_{2}>20\;\mathrm{mmHg}$, ICP<22  mmHg, CPP>60  mmHg) does not guarantee that ischemic conditions are not occuring.^112^ Finally, many patients are not candidates for invasive monitor placement. Non-invasive global measures of physiology (e.g., MAP, ${\mathrm{ETCO}}_{2}$) cannot reliably reflect cerebral physiology. Given the high temporal resolution it offers, real-time non-invasive optical detection of critically-low tissue oxyenation levels via NIRS could facilitate meaningful clinical interventions and improve patient outcomes. In isolation, ${\mathrm{rSO}}_{2}$ does not adequately measure tissue ischemia.^113^ However, in combination with cortical CBF indices (via optical technology, such as DCS)^114^^–^^116^ and arterial oxygen saturation, the oxygen extraction fraction and cerebral metabolic rate of oxygen can be determined and thereby can identify areas of perfusion-metabolic mismatch.^115^^,^^117^ This is a promising area for further studies in neurological disease states.

### 5.3 Non-Invasive Detection of CSDs

Prior studies in pre-clinical models of TBI have demonstrated that not all neuronal damage occurs at the onset of trauma (the primary injury). Instead, subsequent, poorly-understood molecular and cellular mechanisms of secondary injury lead to ischemia and neurotoxicity, which are detrimental to functional outcomes following TBI.^118^ CSDs, first described by Leão^119^ as “cortical spreading depression”, represent one such potential mechanism. CSDs are slowly propagating (1 to 6  mm/min) waves of extreme depolarization followed by suppression of brain activity, which are common after acquired brain injury, including subarachnoid hemorrhage,^120^ ischemic stroke,^121^ and TBI.^122^ CSDs in TBI are notably associated with worse patient outcomes,^123^ and given their delayed nature, offer an appealing therapeutic target for prevention of secondary brain injury. However, real-time monitoring is needed to detect CSDs and titrate potential therapies; given that scalp electroencephalography (EEG) has not proven a reliable diagnostic tool for CSDs,^124^ their detection currently requires subdural electrocorticography. These challenges highlight the need for a non-invasive CSD detection method.

Thought to result from mismatch between energy supply (e.g., CBF, metabolic substrates) and demand (metabolic rate),^125^ CSDs are associated with dramatic changes in neuronal and neurovascular function.^55^^,^^126^ In metabolically intact tissue, spreading depolarizations generally induce vasodilation. However, when the cerebral vasculature is compromised, CSDs commonly trigger a bimodal vascular response, consisting of initial vasoconstriction (i.e., inverse neurovascular coupling) resulting in reduced blood oxygenation, tissue hypoxia, and metabolic failure, followed by vasodilation. It is hypothesized that the deleterious effects of CSDs are in part due to these associated vascular manifestations.^55^ NIRS has been used to non-invasively detect neurovascular changes associated with CSD in migraines,^127^^,^^128^ ischemia in stroke,^25^ as well as cerebral dysregulation in TBI patients.^21^ CSDs produce dramatic changes in neurovascular dynamics, which have the potential to be detected via regional changes in CBF and metabolism as measured by NIRS. This is supported by studies using NIRS to detect spontaneous vascular changes associated with migrainous auras, which are widely thought to be secondary to CSDs.^127^ Because NIRS methods are non-invasive and compatible with continuous bedside monitoring in critically ill patients, this technology represents potential diagnostic and thereapeutic utility in the ICU.

### 5.4 Combined fNIRS and EEG

Combining fNIRS with EEG, which evaluates the excitatory and inhibitory post-synaptic potentials generated from regional neural activity, offers the ability to evaluate neurovascular coupling. Although EEG and fNIRS have been used to study various neurological disorders, including seizures/epilepsy (e.g., delineating non-epileptic events from seizures^129^) and stroke (e.g., response to neurotherapeutic approaches in neurorehabilitation),^54^^,^^130^^–^^132^ there has been limited use in neurocritical care patients. However, given the important role of altered neurovascular coupling in acute forms of brain injury,^54^^,^^55^ the ability to detect and better understand this pathophysiology would be valuable in the ICU. Early studies in NHP models have demonstrated that there is a potential relationship between CPP/autoregulation and neurovascular coupling.^133^ In an NHP communicating hydrocephalus model, exogenous CSF was introducted into the animals ventricles, with CPP measured at each CSF volume. Concurrent EEG and fNIRS were applied to measure neural and vascular, respectively, evoked potentials during a visual stimulus exposure, and a resulting hemodynamic response function (HRF) was dervied as a measure of neurovascular coupling. As CPP became more deranged from physiologic values, the shape of the HRF, and therefore the status of neurovascular coupling, became more altered, independent from the subject’s ${\mathrm{ETCO}}_{2}$.^133^ A better understanding of this mechanism and extension of these EEG-NIRS studies into ICU patients could allow for better CPP optimization post-brain injury.

### 5.5 Quantifying Patient/Caregiver Interactions and Identifying Covert Consciousness

fNIRS has been applied to social interactions in what is termed “hyperscanning.” Classically, this involves a dyad (e.g., parent-child or two competitors in a game) undergoing simultaneous fNIRS recordings, with one instrument’s optodes divided between paricipants. This allows simultaneous interpersonal interactions and their resulting hemodynamic changes to be evaluated in real-time.^134^ Synchronized patterns provide information about various social neuroscience areas of interest, including competition and deception,^135^^,^^136^ cooperation,^137^^,^^138^ group communication,^139^ and childhood socialization/bonding.^140^ A potential future application of hyperscanning in the NICU lies in assessing patient-family/provider interactions in cases of post-injury coma. Given the “black box” that the comatose brain represents, there is much interest in identifying any covert consciousness that may be present. It is conceiveable that patients with better prognosis for functional recovery may have subtle synchronization descovered with concurrent patient-family or patient-provider fNIRS recordings. This would offer assistance with neuroprognostication and therefore guidance to family members.

## 6 Conclusion

NIRS is emerging as a tool that can non-invasively monitor brain function and impact therapeutic decisions for patients admitted to the ICU with acute brain injuries. A growing body of literature suggests roles for identifying patients that develop or are at risk for developing secondary brain injury as well as optimizing hemodynamics based on an individual patient’s autoregulation status. Moreover, recent work in NIRS offers the possibility to non-invasively measure ICP and CrCP to provide important intervenable physiologic metrics.^13^^,^^109^ Advanced NIRS techniques, such as DCS, enable real-time measurements of absolute CBF^141^ and cerebral metabolism^142^ as well as improved ability to define areas of true cerebral ischemia.^116^ In addition, combining NIRS with other bedside tools, such as EEG, may improve its diagnostic accuracy.^143^ Future studies will be required to validate the impact of NIRS on patient outcomes. Finally, evaluating inter-brain synchronization using fNIRS hyperscanning may enable the study of otherwise unmeasurable cognitive interactions between unresponsive patients in the ICU and persons that interact with them (such as family members/caregivers and health care providers).^144^^,^^145^ Given its advantages of portability, non-invasiveness, and low-cost, NIRS will likely become more widely utilized by intensivists in the future.
